# *N*,*N*′-Bis[(*E*)-2-thienylmethyl­ene]-4,4′-oxydianiline

**DOI:** 10.1107/S1600536809033285

**Published:** 2009-08-26

**Authors:** Xuquan Tao, Hui Cui

**Affiliations:** a College of Materials Science and Engineering, Liaocheng University, Shandong 252059, People’s Republic of China; b College of Chemistry and Chemical Engineering, Liaocheng University, Shandong 252059, People’s Republic of China

## Abstract

In the title mol­ecule, C_22_H_16_N_2_OS_2_, which demonstrates non-crystallographic *C*_2_ pseudosymmetry [C—O—C angle = 121.0 (3)°], the two benzene rings make a dihedral angle of 62.09 (14)°. The crystal packing exhibits no significantly short inter­molecular contacts.

## Related literature

For general background, see: Nakajima *et al.* (1998 ▶); Opstal & Verpoort (2003 ▶); Chakraborty & Patel (1996 ▶). For a related structure, see Hu *et al.* (2008 ▶).
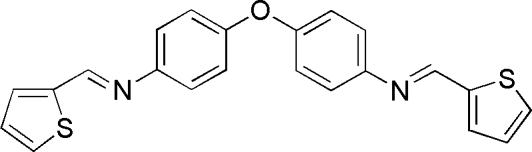


## Experimental

### 

#### Crystal data

C_22_H_16_N_2_OS_2_
*M* *_r_* = 388.49
Monoclinic, 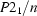

*a* = 6.0897 (7) Å
*b* = 41.478 (3) Å
*c* = 7.5300 (12) Å
β = 90.130 (1)°
*V* = 1902.0 (4) Å^3^
*Z* = 4
Mo *K*α radiation
μ = 0.29 mm^−1^
*T* = 298 K
0.40 × 0.37 × 0.05 mm

#### Data collection

Bruker SMART APEX CCD area-detector diffractometer
Absorption correction: multi-scan (*SADABS*; Sheldrick, 1996 ▶) *T*_min_ = 0.891, *T*_max_ = 0.985
8674 measured reflections
3319 independent reflections
2079 reflections with *I* > 2σ(*I*)
*R*_int_ = 0.073

#### Refinement

*R*[*F* ^2^ > 2σ(*F* ^2^)] = 0.090
*wR*(*F* ^2^) = 0.218
*S* = 1.08
3319 reflections
244 parameters
H-atom parameters constrained
Δρ_max_ = 0.26 e Å^−3^
Δρ_min_ = −0.36 e Å^−3^

### 

Data collection: *SMART* (Siemens, 1996 ▶); cell refinement: *SAINT* (Siemens, 1996 ▶); data reduction: *SAINT*; program(s) used to solve structure: *SHELXS97* (Sheldrick, 2008 ▶); program(s) used to refine structure: *SHELXL97* (Sheldrick, 2008 ▶); molecular graphics: *SHELXTL* (Sheldrick, 2008 ▶); software used to prepare material for publication: *SHELXTL*.

## Supplementary Material

Crystal structure: contains datablocks I, global. DOI: 10.1107/S1600536809033285/cv2595sup1.cif

Structure factors: contains datablocks I. DOI: 10.1107/S1600536809033285/cv2595Isup2.hkl

Additional supplementary materials:  crystallographic information; 3D view; checkCIF report

## Figures and Tables

**Fig. 1.**
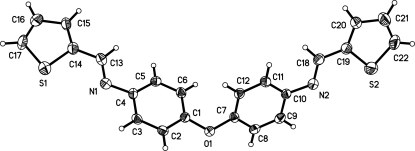
The molecular structure of (I) showing the atomic numbering scheme and 30% probability displacement ellipsoids.

## supplementary crystallographic information

### Comment

In the recent years, there is a considerable interest in the chemistry of Schiff bases (Nakajima *et al.*, 1998). This is due to the fact that Schiff bases offer opportunities for inducing substrate chirality, tuning the metal centred electronic factor, enhancing the solubility and stability of either homogeneous or heterogeneous catalysts (Opstal & Verpoort, 2003). Schiff base complexes with metals exhibit strong anticancer activity (Chakraborty & Patel, 1996). Here, we report the synthesis and crystal structure of the title compound (I)- new flexible Schiff-base ligand.

The molecule of (I) is shown in Fig. 1. Bond lengths and angles are comparable with those observed in similar compounds (Hu *et al.*, 2008). The C13=N1 and C18=N2 bond lengths of 1.244 (6) and 1.253 (6) Å, respectively, are usual for C=N double bond. Each half of the molecule displays a *trans* configuration across the C=N double bond. The dihedral angles between the benzene rings C1-C6 and C7-C12 is 62.09 (14) °.

In the crystal structure, there are no significantly short intermolecular contacts.

### Experimental

4-(4^'^-Aminophenoxy)benzenamine (10 mmol), thiophene-2-carbaldehyde (20 mmol) and 20 ml of ethanol were mixed in 50 ml flask. After stirring for 3h at 303 K, the resulting mixture was recrystallized from ethanol, affording the title compound as orange crystalline solid.

### Refinement

All H atoms were placed in geometrically idealized positions (C—H 0.93 Å) and treated as riding on their parent atoms, with *U*_iso_(H) = 1.2 *U*_eq_(C).

### Crystal data

**Table:**

C_22_H_16_N_2_OS_2_	*F*(000) = 808
*M**_r_* = 388.49	*D*_x_ = 1.357 Mg m^−^^3^
Monoclinic, *P*2_1_/*n*	Mo *K*α radiation, λ = 0.71073 Å
*a* = 6.0897 (7) Å	Cell parameters from 2059 reflections
*b* = 41.478 (3) Å	θ = 3.9–25.0°
*c* = 7.5300 (12) Å	µ = 0.29 mm^−^^1^
β = 90.130 (1)°	*T* = 298 K
*V* = 1902.0 (4) Å^3^	Block, red
*Z* = 4	0.40 × 0.37 × 0.05 mm

### Data collection

**Table:**

Bruker SMART APEX CCD area-detector diffractometer	3319 independent reflections
Radiation source: fine-focus sealed tube	2079 reflections with *I* > 2σ(*I*)
graphite	*R*_int_ = 0.073
φ and ω scans	θ_max_ = 25.0°, θ_min_ = 2.0°
Absorption correction: multi-scan (*SADABS*; Sheldrick, 1996)	*h* = −6→7
*T*_min_ = 0.891, *T*_max_ = 0.985	*k* = −49→38
8674 measured reflections	*l* = −8→7

### Refinement

**Table:**

Refinement on *F*^2^	Primary atom site location: structure-invariant direct methods
Least-squares matrix: full	Secondary atom site location: difference Fourier map
*R*[*F*^2^ > 2σ(*F*^2^)] = 0.090	Hydrogen site location: inferred from neighbouring sites
*wR*(*F*^2^) = 0.218	H-atom parameters constrained
*S* = 1.08	*w* = 1/[σ^2^(*F*_o_^2^) + (0.0631*P*)^2^ + 3.3823*P*] where *P* = (*F*_o_^2^ + 2*F*_c_^2^)/3
3319 reflections	(Δ/σ)_max_ < 0.001
244 parameters	Δρ_max_ = 0.26 e Å^−^^3^
0 restraints	Δρ_min_ = −0.36 e Å^−^^3^

### Special details

**Table:**

Geometry. All e.s.d.'s (except the e.s.d. in the dihedral angle between two l.s. planes) are estimated using the full covariance matrix. The cell e.s.d.'s are taken into account individually in the estimation of e.s.d.'s in distances, angles and torsion angles; correlations between e.s.d.'s in cell parameters are only used when they are defined by crystal symmetry. An approximate (isotropic) treatment of cell e.s.d.'s is used for estimating e.s.d.'s involving l.s. planes.
Refinement. Refinement of *F*^2^ against ALL reflections. The weighted *R*-factor *wR* and goodness of fit *S* are based on *F*^2^, conventional *R*-factors *R* are based on *F*, with *F* set to zero for negative *F*^2^. The threshold expression of *F*^2^ > σ(*F*^2^) is used only for calculating *R*-factors(gt) *etc*. and is not relevant to the choice of reflections for refinement. *R*-factors based on *F*^2^ are statistically about twice as large as those based on *F*, and *R*- factors based on ALL data will be even larger.

### Fractional atomic coordinates and isotropic or equivalent isotropic displacement parameters (Å^2^)

**Table:**

	*x*	*y*	*z*	*U*_iso_*/*U*_eq_	
S1	0.4955 (3)	0.05770 (4)	0.6056 (3)	0.0906 (6)	
S2	0.4773 (3)	0.44248 (5)	0.5080 (3)	0.0998 (7)	
N1	0.6149 (7)	0.12778 (10)	0.5599 (6)	0.0552 (11)	
N2	0.6082 (7)	0.37230 (10)	0.5480 (6)	0.0529 (11)	
O1	0.9917 (5)	0.25041 (8)	0.5517 (5)	0.0489 (8)	
C1	0.8811 (7)	0.22122 (11)	0.5554 (6)	0.0409 (11)	
C2	0.9821 (7)	0.19597 (11)	0.6410 (6)	0.0443 (12)	
H2	1.1137	0.1994	0.7010	0.053*	
C3	0.8919 (7)	0.16597 (11)	0.6389 (6)	0.0450 (12)	
H3	0.9637	0.1492	0.6969	0.054*	
C4	0.6937 (8)	0.15984 (10)	0.5516 (6)	0.0419 (11)	
C5	0.5935 (8)	0.18563 (11)	0.4622 (6)	0.0470 (12)	
H5	0.4627	0.1822	0.4012	0.056*	
C6	0.6854 (8)	0.21602 (11)	0.4629 (6)	0.0451 (12)	
H6	0.6177	0.2329	0.4024	0.054*	
C7	0.8781 (7)	0.27942 (10)	0.5519 (6)	0.0397 (11)	
C8	0.9794 (8)	0.30454 (12)	0.4627 (6)	0.0457 (12)	
H8	1.1085	0.3009	0.4001	0.055*	
C9	0.8885 (8)	0.33485 (12)	0.4669 (6)	0.0475 (12)	
H9	0.9595	0.3519	0.4109	0.057*	
C10	0.6885 (7)	0.34028 (11)	0.5557 (6)	0.0412 (11)	
C11	0.5918 (8)	0.31457 (11)	0.6446 (6)	0.0473 (12)	
H11	0.4615	0.3178	0.7062	0.057*	
C12	0.6863 (7)	0.28442 (11)	0.6428 (6)	0.0440 (11)	
H12	0.6201	0.2675	0.7032	0.053*	
C13	0.4187 (9)	0.12173 (12)	0.5275 (7)	0.0558 (14)	
H13	0.3245	0.1384	0.4960	0.067*	
C14	0.3329 (9)	0.08851 (12)	0.5382 (7)	0.0556 (14)	
C15	0.1330 (9)	0.07893 (13)	0.4965 (8)	0.0655 (16)	
H15	0.0224	0.0927	0.4570	0.079*	
C16	0.1061 (11)	0.04474 (15)	0.5191 (10)	0.085 (2)	
H16	−0.0240	0.0338	0.4956	0.102*	
C17	0.2895 (12)	0.03046 (15)	0.5779 (10)	0.084 (2)	
H17	0.3025	0.0085	0.6006	0.101*	
C18	0.4106 (9)	0.37824 (12)	0.5808 (7)	0.0555 (13)	
H18	0.3178	0.3613	0.6101	0.067*	
C19	0.3211 (9)	0.41082 (12)	0.5748 (7)	0.0553 (13)	
C20	0.1186 (9)	0.42042 (13)	0.6180 (8)	0.0623 (15)	
H20	0.0087	0.4065	0.6558	0.075*	
C21	0.0893 (11)	0.45432 (15)	0.6002 (9)	0.081 (2)	
H21	−0.0408	0.4650	0.6270	0.097*	
C22	0.2677 (13)	0.46897 (15)	0.5412 (12)	0.100 (3)	
H22	0.2775	0.4910	0.5203	0.120*	

### Atomic displacement parameters (Å^2^)

**Table:**

	*U*^11^	*U*^22^	*U*^33^	*U*^12^	*U*^13^	*U*^23^
S1	0.0697 (11)	0.0664 (11)	0.1356 (17)	0.0015 (9)	−0.0133 (10)	0.0213 (11)
S2	0.0662 (11)	0.0620 (11)	0.171 (2)	−0.0033 (9)	0.0046 (11)	0.0270 (11)
N1	0.049 (3)	0.052 (3)	0.064 (3)	0.002 (2)	−0.002 (2)	0.004 (2)
N2	0.050 (3)	0.045 (3)	0.064 (3)	−0.001 (2)	0.001 (2)	0.001 (2)
O1	0.0408 (17)	0.0418 (18)	0.064 (2)	−0.0024 (15)	0.0016 (15)	−0.0023 (15)
C1	0.041 (3)	0.040 (3)	0.042 (3)	0.000 (2)	0.003 (2)	−0.001 (2)
C2	0.038 (3)	0.047 (3)	0.047 (3)	0.005 (2)	−0.004 (2)	−0.001 (2)
C3	0.044 (3)	0.045 (3)	0.046 (3)	0.008 (2)	−0.006 (2)	0.007 (2)
C4	0.049 (3)	0.034 (3)	0.043 (3)	0.000 (2)	0.004 (2)	0.002 (2)
C5	0.042 (3)	0.049 (3)	0.050 (3)	−0.003 (2)	−0.012 (2)	0.000 (2)
C6	0.049 (3)	0.041 (3)	0.045 (3)	0.005 (2)	−0.008 (2)	0.004 (2)
C7	0.045 (3)	0.037 (3)	0.038 (3)	0.003 (2)	−0.005 (2)	−0.003 (2)
C8	0.038 (3)	0.052 (3)	0.047 (3)	−0.008 (2)	0.002 (2)	−0.005 (2)
C9	0.048 (3)	0.047 (3)	0.048 (3)	−0.011 (2)	−0.001 (2)	0.006 (2)
C10	0.041 (3)	0.040 (3)	0.043 (3)	−0.001 (2)	−0.005 (2)	−0.002 (2)
C11	0.045 (3)	0.046 (3)	0.050 (3)	0.001 (2)	0.010 (2)	−0.001 (2)
C12	0.044 (3)	0.041 (3)	0.046 (3)	−0.004 (2)	0.008 (2)	0.001 (2)
C13	0.061 (4)	0.047 (3)	0.059 (3)	0.010 (3)	−0.001 (3)	−0.002 (2)
C14	0.062 (3)	0.047 (3)	0.058 (3)	0.001 (3)	0.006 (3)	0.000 (2)
C15	0.061 (4)	0.038 (3)	0.097 (5)	0.001 (3)	−0.022 (3)	0.002 (3)
C16	0.062 (4)	0.063 (4)	0.131 (7)	−0.015 (3)	−0.002 (4)	−0.009 (4)
C17	0.092 (5)	0.041 (4)	0.118 (6)	−0.009 (3)	0.022 (4)	0.013 (3)
C18	0.053 (3)	0.051 (3)	0.062 (3)	−0.007 (3)	−0.003 (3)	0.001 (3)
C19	0.055 (3)	0.050 (3)	0.061 (3)	−0.002 (3)	−0.006 (3)	0.001 (3)
C20	0.057 (3)	0.051 (3)	0.079 (4)	0.000 (3)	0.012 (3)	−0.005 (3)
C21	0.072 (4)	0.066 (4)	0.103 (5)	0.020 (4)	−0.018 (4)	−0.009 (4)
C22	0.089 (5)	0.041 (4)	0.170 (8)	−0.005 (4)	−0.021 (5)	0.006 (4)

### Geometric parameters (Å, °)

**Table:**

S1—C14	1.694 (5)	C8—H8	0.9300
S1—C17	1.701 (7)	C9—C10	1.409 (7)
S2—C19	1.698 (5)	C9—H9	0.9300
S2—C22	1.703 (8)	C10—C11	1.390 (6)
N1—C13	1.244 (6)	C11—C12	1.377 (6)
N1—C4	1.415 (6)	C11—H11	0.9300
N2—C18	1.253 (6)	C12—H12	0.9300
N2—C10	1.416 (6)	C13—C14	1.476 (7)
O1—C1	1.386 (5)	C13—H13	0.9300
O1—C7	1.388 (5)	C14—C15	1.318 (7)
C1—C2	1.374 (6)	C15—C16	1.438 (8)
C1—C6	1.396 (6)	C15—H15	0.9300
C2—C3	1.361 (6)	C16—C17	1.338 (9)
C2—H2	0.9300	C16—H16	0.9300
C3—C4	1.396 (6)	C17—H17	0.9300
C3—H3	0.9300	C18—C19	1.458 (7)
C4—C5	1.403 (6)	C18—H18	0.9300
C5—C6	1.379 (6)	C19—C20	1.337 (7)
C5—H5	0.9300	C20—C21	1.423 (8)
C6—H6	0.9300	C20—H20	0.9300
C7—C12	1.371 (6)	C21—C22	1.323 (9)
C7—C8	1.385 (6)	C21—H21	0.9300
C8—C9	1.374 (6)	C22—H22	0.9300
			
C14—S1—C17	91.9 (3)	C12—C11—H11	119.5
C19—S2—C22	92.0 (3)	C10—C11—H11	119.5
C13—N1—C4	120.4 (4)	C7—C12—C11	119.9 (4)
C18—N2—C10	120.5 (4)	C7—C12—H12	120.0
C1—O1—C7	121.0 (3)	C11—C12—H12	120.0
C2—C1—O1	117.3 (4)	N1—C13—C14	121.2 (5)
C2—C1—C6	119.8 (4)	N1—C13—H13	119.4
O1—C1—C6	122.6 (4)	C14—C13—H13	119.4
C3—C2—C1	120.8 (4)	C15—C14—C13	126.5 (5)
C3—C2—H2	119.6	C15—C14—S1	112.5 (4)
C1—C2—H2	119.6	C13—C14—S1	120.9 (4)
C2—C3—C4	121.4 (4)	C14—C15—C16	112.0 (5)
C2—C3—H3	119.3	C14—C15—H15	124.0
C4—C3—H3	119.3	C16—C15—H15	124.0
C3—C4—C5	117.5 (4)	C17—C16—C15	112.4 (6)
C3—C4—N1	116.3 (4)	C17—C16—H16	123.8
C5—C4—N1	126.2 (4)	C15—C16—H16	123.8
C6—C5—C4	121.3 (4)	C16—C17—S1	111.2 (5)
C6—C5—H5	119.4	C16—C17—H17	124.4
C4—C5—H5	119.4	S1—C17—H17	124.4
C5—C6—C1	119.3 (4)	N2—C18—C19	122.3 (5)
C5—C6—H6	120.4	N2—C18—H18	118.8
C1—C6—H6	120.4	C19—C18—H18	118.8
C12—C7—C8	120.6 (4)	C20—C19—C18	127.9 (5)
C12—C7—O1	123.8 (4)	C20—C19—S2	111.1 (4)
C8—C7—O1	115.4 (4)	C18—C19—S2	121.1 (4)
C9—C8—C7	119.8 (4)	C19—C20—C21	112.8 (5)
C9—C8—H8	120.1	C19—C20—H20	123.6
C7—C8—H8	120.1	C21—C20—H20	123.6
C8—C9—C10	120.4 (4)	C22—C21—C20	112.5 (6)
C8—C9—H9	119.8	C22—C21—H21	123.8
C10—C9—H9	119.8	C20—C21—H21	123.8
C11—C10—C9	118.2 (4)	C21—C22—S2	111.7 (5)
C11—C10—N2	126.3 (4)	C21—C22—H22	124.2
C9—C10—N2	115.4 (4)	S2—C22—H22	124.2
C12—C11—C10	121.0 (4)		
			
C7—O1—C1—C2	−148.1 (4)	N2—C10—C11—C12	−179.8 (4)
C7—O1—C1—C6	38.0 (7)	C8—C7—C12—C11	0.5 (7)
O1—C1—C2—C3	−175.0 (4)	O1—C7—C12—C11	175.9 (4)
C6—C1—C2—C3	−1.0 (7)	C10—C11—C12—C7	−0.2 (7)
C1—C2—C3—C4	−0.5 (7)	C4—N1—C13—C14	−179.1 (4)
C2—C3—C4—C5	1.5 (7)	N1—C13—C14—C15	−175.5 (6)
C2—C3—C4—N1	−179.4 (4)	N1—C13—C14—S1	3.3 (7)
C13—N1—C4—C3	161.5 (5)	C17—S1—C14—C15	−0.1 (5)
C13—N1—C4—C5	−19.6 (8)	C17—S1—C14—C13	−179.0 (5)
C3—C4—C5—C6	−1.1 (7)	C13—C14—C15—C16	178.8 (6)
N1—C4—C5—C6	−180.0 (5)	S1—C14—C15—C16	−0.1 (7)
C4—C5—C6—C1	−0.4 (7)	C14—C15—C16—C17	0.2 (9)
C2—C1—C6—C5	1.4 (7)	C15—C16—C17—S1	−0.3 (8)
O1—C1—C6—C5	175.2 (4)	C14—S1—C17—C16	0.2 (6)
C1—O1—C7—C12	35.5 (6)	C10—N2—C18—C19	179.9 (5)
C1—O1—C7—C8	−148.9 (4)	N2—C18—C19—C20	−175.2 (6)
C12—C7—C8—C9	0.8 (7)	N2—C18—C19—S2	4.4 (8)
O1—C7—C8—C9	−175.0 (4)	C22—S2—C19—C20	0.6 (5)
C7—C8—C9—C10	−2.5 (7)	C22—S2—C19—C18	−179.1 (5)
C8—C9—C10—C11	2.8 (7)	C18—C19—C20—C21	178.5 (5)
C8—C9—C10—N2	−178.7 (4)	S2—C19—C20—C21	−1.1 (7)
C18—N2—C10—C11	−19.9 (7)	C19—C20—C21—C22	1.2 (9)
C18—N2—C10—C9	161.6 (5)	C20—C21—C22—S2	−0.8 (9)
C9—C10—C11—C12	−1.4 (7)	C19—S2—C22—C21	0.1 (6)
